# Synthesis, In Vitro Biological Evaluation and Molecular Modeling of Benzimidazole-Based Pyrrole/Piperidine Hybrids Derivatives as Potential Anti-Alzheimer Agents

**DOI:** 10.3390/ph17040410

**Published:** 2024-03-24

**Authors:** Sundas Tariq, Fazal Rahim, Hayat Ullah, Maliha Sarfraz, Rafaqat Hussain, Shoaib Khan, Misbah Ullah Khan, Wajid Rehman, Amjad Hussain, Mashooq Ahmad Bhat, Muhammad Kamran Farooqi, Syed Adnan Ali Shah, Naveed Iqbal

**Affiliations:** 1 Department of Chemistry, Hazara University, Mansehra 21120, Pakistan; sundastariqkhan1993@gmail.com (S.T.); rafaqathussain0347@gmail.com (R.H.); sono_wej@yahoo.com (W.R.); 2 Institute of Chemistry, University of Okara, Okara 56130, Pakistan; amjadhussain@uo.edu.pk; 3 Department of Zoology, Wildlife and Fisheries, University of Agriculture Faisalabad, Sub-Campus Toba Tek Singh, Toba Tek Singh 36080, Pakistan; maliha.sarfraz@uaf.edu.pk; 4 Department of Chemistry, Abbottabad University of Science and Technology, Abbottabad 22500, Pakistan; shoaibkhanswati@gmail.com; 5 Center for Nanosciences, University of Okara, Okara 56130, Pakistan; misbahullahkhan143@uo.edu.pk; 6 Department of Pharmaceutical Chemistry, College of Pharmacy, King Saud University, Riyadh 11451, Saudi Arabia; mabhat@ksu.edu.sa; 7 Hubei Key Laboratory of Material Chemistry and Service Failure, School of Chemistry and Chemical Engineering, Huazhong University of Science and Technology, 1037 Luoyu Road, Hongshan District, Wuhan 430074, China; kamranchemstry096@gmail.com; 8 Faculty of Pharmacy, Universiti Teknologi MARA Cawangan Selangor Kampus Puncak Alam, Bandar Puncak Alam 42300, Selangor, Malaysia; syedadnan@utm.edu.my; 9 Atta-ur-Rahman Institute for Natural Product Discovery (AuRIns), Universiti Teknologi MARA Cawangan Selangor Kampus Puncak Alam, Bandar Puncak Alam 42300, Selangor, Malaysia; 10 Department of Chemistry, University of Poonch, Rawalakot 12350, Pakistan; naveediqbal@upr.edu.pk

**Keywords:** synthesis, benzimidazole, pyrrole, piperidine, AChE and BuChE and molecular docking

## Abstract

Benzimidazole-based pyrrole/piperidine analogs (**1**–**26**) were synthesized and then screened for their acetylcholinesterase and butyrylcholinesterase activities. All the analogs showed good to moderate cholinesterase activities. Synthesized compounds (**1**–**13**) were screened in cholinesterase enzyme inhibition assays and showed AChE activities in the range of IC_50_ = 19.44 ± 0.60 µM to 36.05 ± 0.4 µM against allanzanthane (IC_50_ = 16.11 ± 0.33 µM) and galantamine (IC_50_ = 19.34 ± 0.62 µM) and varied BuChE inhibitory activities, with IC_50_ values in the range of 21.57 ± 0.61 µM to 39.55 ± 0.03 µM as compared with standard allanzanthane (IC_50_ = 18.14 ± 0.05 µM) and galantamine (IC_50_ = 21.45 ± 0.21 µM). Similarly, synthesized compounds (**14**–**26**) were also subjected to tests to determine their in vitro AChE inhibitory activities, and the results obtained corroborated that all the compounds showed varied activities in the range of IC_50_ = 22.07 ± 0.13 to 42.01 ± 0.02 µM as compared to allanzanthane (IC_50_ = 20.01 ± 0.12 µM) and galantamine (IC_50_ = 18.05 ± 0.31 µM) and varied BuChE inhibitory activities, with IC_50_ values in the range of 26.32 ± 0.13 to 47.03 ± 0.15 µM as compared to standard allanzanthane (IC_50_ = 18.14 ± 0.05 µM) and galantamine (IC_50_ = 21.45 ± 0.21 µM). Binding interactions of the most potent analogs were confirmed through molecular docking studies. The active analogs **2**, **4**, **10** and **13** established numerous interactions with the active sites of targeted enzymes, with docking scores of −10.50, −9.3, −7.73 and −7.8 for AChE and −8.97, −8.2, −8.20 and −7.6 for BuChE, respectively.

## 1. Introduction

**Figure 1:**
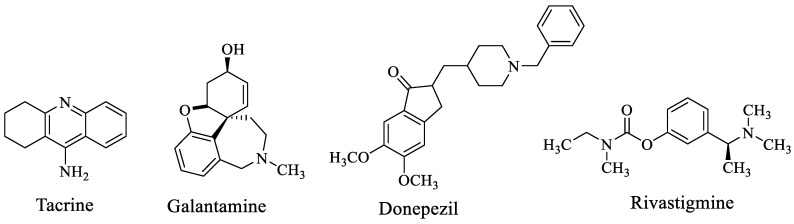
Available drugs for AD with AChE activity.

Alzheimer’s disease (AD) is a chronic neurological disorder characterized by memory impairment, cognitive dysfunction, behavioral disturbances and deficits in activities of daily living [1,2,3]. AD has been found to be associated with a cholinergic deficit in the postmortem brain characterized by a significant decrease in the amount of acetylcholine [4,5]. AD has become a major problem, particularly in developed countries, due to increasing old-age populations with a high quality of life. Acetylcholine is a neurotransmitter inhibited, primarily, by acetylcholinesterase (AChE) and, secondarily, by butyrylcholinesterase (BuChE), which are considered to play a role in the pathology of AD [6]. Both enzymes are present in the brain and are detected in neurofibrillary tangles and neurotic plaques [7]. Despite the unknown etiology of AD, elevation of acetylcholine amounts through AChE enzyme inhibition has been accepted as the most effective treatment strategy against AD [8]. Therefore, AChE and BuChE inhibitors have become remarkable alternatives in the treatment of AD. However, the present drugs (e.g., tacrine, galantamine, donepezil and rivastigmine) (Figure 1) with AChE inhibitory activity possess some side effects and are effective only against the mild type of AD, and currently there is no drug with BuChE inhibitory activity available [9,10].

**Figure 2:**
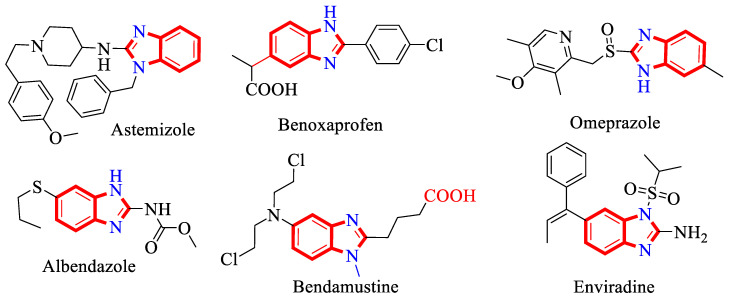
Benzimidazole analogs bearing bioactive drugs.

Benzimidazole possesses a diverse range of biological activities, such as anti-hypertensive [11,12], anti-Alzheimer’s [13], anti-microbial, anti-viral [14], anti-diabetic [15,16] and anti-cancer activities [17]. There are some drug molecules, such as benoxaprofen, albendazole, enviradine, bendamastine, omeprazole and astemizole, which contain a benzimidazole moiety in their skeleton (Figure 2) [18]. 

Pyrrole derivatives have been found to exhibit a wide range of biological activities, including anti-bacterial, anti-fungal, anti-viral, anti-inflammatory, analgesic, anti-tumor and anti-convulsant activities. Pyrrole-containing drugs, such as atorvastatin, are used to treat hypercholesterolemia [19], and tolmetin is used to treat osteoarthritis and rheumatoid arthritis [20]. Nargenicin is an antibacterial drug that contains a pyrrole ring [21]. Prodigiosin is an anti-cancer compound that contains three pyrrole scaffolds [22]. Piperidine and its derivatives have great biological significance in medicinal chemistry and are widely used in the development of drugs for the treatment of various diseases. Their unique chemical properties and ability to interact with a variety of biological targets make them important scaffolds for drug design [23], including anti-cancer drugs [24,25,26,27,28], antibiotics [29], analgesics [30], antidepressants [31] and antioxidants [32]. Keeping in view the biological significance of benzimidazole, pyrrole and piperidine moieties, in this study, we designed and synthesized hybrid scaffolds of benzimidazole-bearing pyrroles and piperidines as potential inhibitors of AChE and BuChE for the treatment of Alzheimer’s disease in the search for lead molecules, and the results obtained corroborated that these compounds could be considered as potential lead molecules for the development of improved AChE and BuChE inhibitors.

## 2. Results and Discussion 

### 2.1. Chemistry

**Scheme 1:**
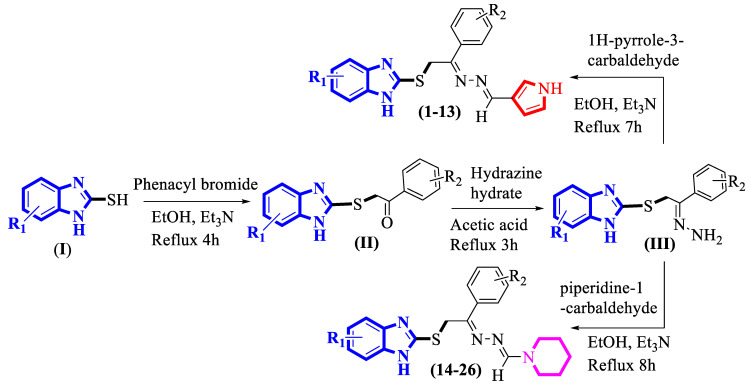
Synthesis of targeted benzimidazole-based hybrid compounds (**1**–**26**).

The synthesis of targeted benzimidazole-based pyrrole/piperidine derivatives (**1**–**26**) was achieved in a series of steps. Initially, 2-marcaptobenzimidazole (**I**) was reacted with differently substituted phenacyl bromides in ethanol and triethylamine, and then a reaction mixture was refluxed for 4 h to give S-substituted benzimidazole (**II**), which was further redissolved in a hydrazine hydrate solution while being stirred in acetic acid. After being refluxed for 3 h, the solvent was evaporated, and the resulting solid residue (**III**) was reacted in two different ways:

(1) Substrate (**III**) was put in ethanol and triethylamine, followed by the addition of 1H-pyrrole-3-carbaldehyde. The reaction mixture was refluxed and stirred for 7 h to obtain targeted benzimidazole-based pyrrole derivatives (**1**–**13**).
(2) Further, the intermediate (**III**) was reacted with piperidine-1-carbaldehyde in ethanol along with a catalytic amount of triethylamine, and the resulting solid residue was heated over a sand bath for 8 h to afford targeted benzimidazole-based piperidine derivatives (**14**–**26**) (Scheme 1).

### 2.2. In Vitro Acetylcholinesterase and Butyrylcholinesterase Activities

The synthesized scaffolds based on benzimidazole-bearing pyrroles/piperidines (**1**–**26**) were subjected to in vitro acetylcholinesterase and butyrylcholinesterase inhibitory activity assays. All the newly synthesized compounds showed good to moderate acetylcholinesterase and butyrylcholinesterase inhibitory activities. Target compounds (**1**–**13**) were screened in assays for cholinesterase enzyme inhibition and showed AChE activities in the range of IC_50_ = 19.44 ± 0.60 µM–36.05 ± 0.4 µM against allanzanthane (IC_50_ = 16.11 ± 0.33 µM) and galantamine (IC_50_ = 19.34 ± 0.62 µM) and varied BuChE inhibitory activities, with IC_50_ values in the range of 21.57 ± 0.61 µM–39.55 ± 0.03 µM. The results were compared with those of standard allanzanthane (IC_50_ = 18.14 ± 0.05 µM) and galantamine (IC_50_ = 21.45 ± 0.21 µM). Similarly, target compounds (**14**–**26**) were also subjected to in vitro AChE inhibitory activity assays, and the results obtained corroborated that all the compounds showed varied activities in the range of IC_50_ = 22.07 ± 0.13–42.01 ± 0.02 µM against allanzanthane (IC_50_ = 20.01 ± 0.12 µM) and galantamine (IC_50_ = 18.05 ± 0.31 µM) and varied BuChE inhibitory activities, with IC_50_ values in the range of 26.32 ± 0.13–47.03 ± 0.15 µM as compared to standard allanzanthane (IC_50_ = 18.14 ± 0.05 µM) and galantamine (IC_50_ = 21.45 ± 0.21 µM). 

#### Structure–Activity Relationship (SAR) Studies for Acetylcholinesterase and Butyrylcholinesterase Activities

By inspecting the influence of various moieties (R_1_ and R_2_) on AChE and BuChE activities, SAR studies were established. Among the series (**1**–**13**), compound **10** (bearing a NO_2_ at the 5-position of the benzimidazole ring and a *m*-NO_2_ substitution on the aryl ring) emerged as the most potent inhibitor of AChE and BuChE enzymes. Furthermore, compound **2** (that has a –OCH_3_ group at the 5-position of the benzimidazole ring and a *p*-OCH_3_ substitution on the aryl ring) also showed great inhibitory potential against the targeted enzymes. However, an alteration in inhibitory potential was observed by changing the position of the –OCH_3_ group around the aryl ring. Therefore, compound **4** (that has a –OCH_3_ group at the 5-position of the benzimidazole ring and a *o*-OCH_3_ substitution on the aryl ring), which holds substituents of a similar nature but has the –OCH_3_ group in a different position on the aryl ring, demonstrated a lesser inhibition profile. Moreover, compound **8** (with an unsubstituted benzimidazole ring and a *o*-OCH_3_ substitutedaryl ring) showed great inhibitory potential when compared to compound **9** (with an unsubstituted benzimidazole ring and *p*-OCH_3_ substituted in the aryl ring), indicating that a change in the position of the substituent around the aryl ring may cause an alteration to the inhibitory potential (Table 1). 

It was noteworthy that analogs (**10**–**13**) bearing a –NO_2_ substituent at the 5-position of the benzimidazole ring and various groups, like –NO_2_, –Ph, –OCH_3_ and –CH_3_, at the variable position of the other aryl ring were identified as encouraging the inhibition of AChE and BuChE enzymes. Among these analogs, analog **10** was shown as a potent inhibitor of the targeted enzymes. Further, the comparison of analog **12** (which has a 5-NO_2_ substitution at the benzimidazole ring and a *p*-OCH_3_ substituted at the aryl ring) with compounds **11** (which has a 5-NO_2_ substitution at the benzimidazole ring and a *p*-Ph moiety at the aryl ring) and **13** (which has a 5-NO_2_ substitution at the benzimidazole ring and a *p*-CH_3_ group on the aryl ring) demonstrated that analog **12** showed a better inhibitory potential than its counterparts. This difference in inhibitory potential may perhaps be owing to the different nature of the attached substituents around the aryl ring, which cause an alteration in the inhibitory potential (Table 1).

Among the series (**14**–**26**), compound **26** (which has a NO_2_ at the 5-position of the benzimidazole ring and a *m*-NO_2_ substitution on the aryl ring) emerged as the most active inhibitor of AChE and BuChE enzymes, followed by compound **17**, bearing a OCH_3_ at the 5-position of the benzimidazole ring and a *m*-NO_2_ substitution on the aryl ring). Compounds **15** and **16**, which have the same 5-OCH_3_ group on the benzimidazole ring and *o*-OCH_3_ and *p*-OCH_3_ substitutions on the aryl ring, also exhibited good inhibition against both AChE and BuChE. When the substituents on the aryl ring were replaced by *p*-CH_3_ and *p*-phenyl in compounds **18** and **14**, respectively, a slight change in their inhibitory activities was observed. Compound **20**, bearing a benzimidazole ring and a *m*-NO_2_ group on the phenyl ring showed good inhibition. When the *m*-NO_2_ group was replaced by *p*-CH_3_, *o*-OCH_3_ and *p*-OCH3 in compounds **19**, **21** and **22**, respectively, a decline in inhibitory activities was observed. Among compounds **23**–**26** (which have the same 5-NO_2_ substitution at the benzimidazole ring but different substituents, like *p*-Ph, *p*-OCH3, *p*-CH_3_ and *m*-NO_2_, at the phenyl ring), compound **26** exhibited significant inhibitory potential, while the remaining three compounds exhibited inhibition levels in the following order: **24** > **25** > **23** (Table 1). The variation in inhibitory potential could be attributed to the distinct characteristics of the substituents attached around the aryl ring, which potentially led to alterations in inhibitory effectiveness.

### 2.3. In Silico Molecular Docking Approach 

#### 2.3.1. Docking Study for Benzimidazole-Based Pyrrole Analogs 

**Figure 3:**
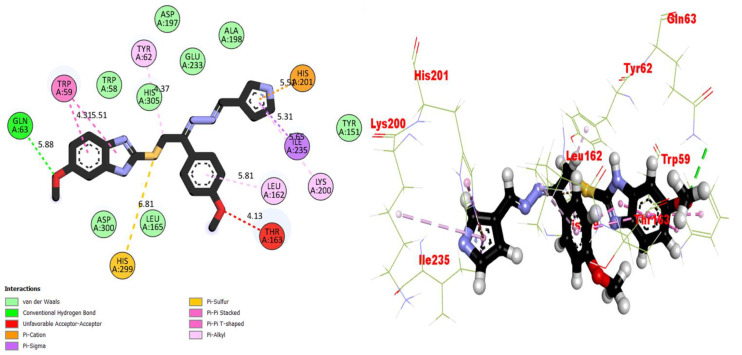
Representations of the protein–ligand interaction (PLI) profile of compound **2** in the AChE complex.

**Figure 4:**
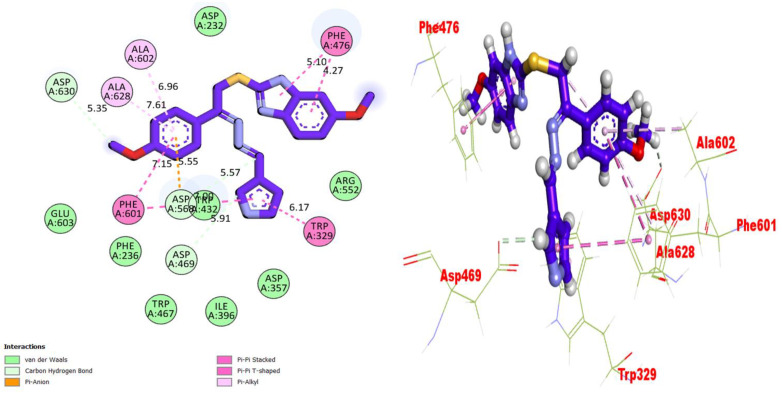
Representations of the protein–ligand interaction (PLI) profile of compound **2** in the BuChE complex.

**Figure 5:**
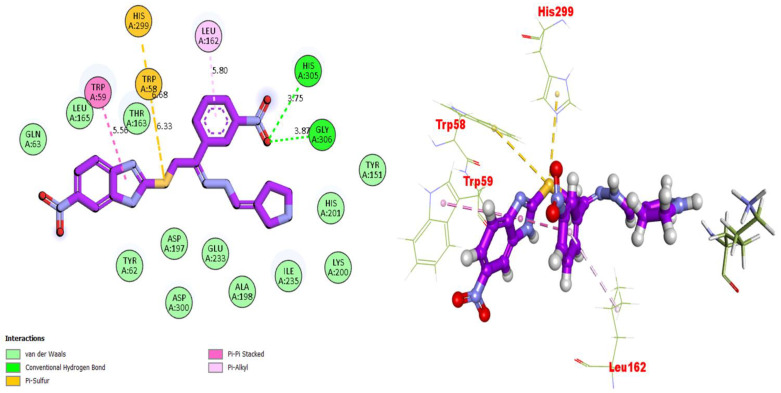
Representations of the protein–ligand interaction (PLI) profile of compound **10** in the AChE complex.

**Figure 6:**
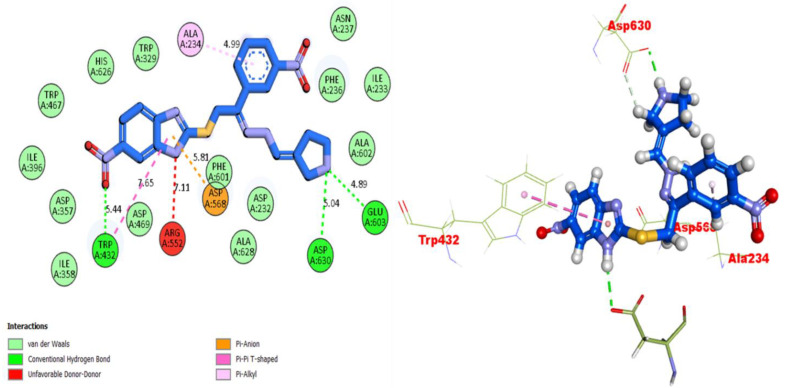
Representations of the protein–ligand interaction (PLI) profile of compound **10** in the BuChE complex.

**Figure 7:**
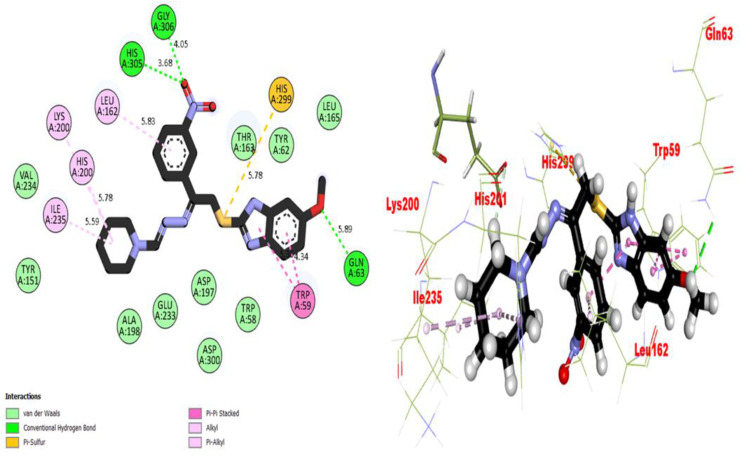
Representations of the protein–ligand interaction (PLI) profile of compound **4** in the AChE complex.

**Figure 8:**
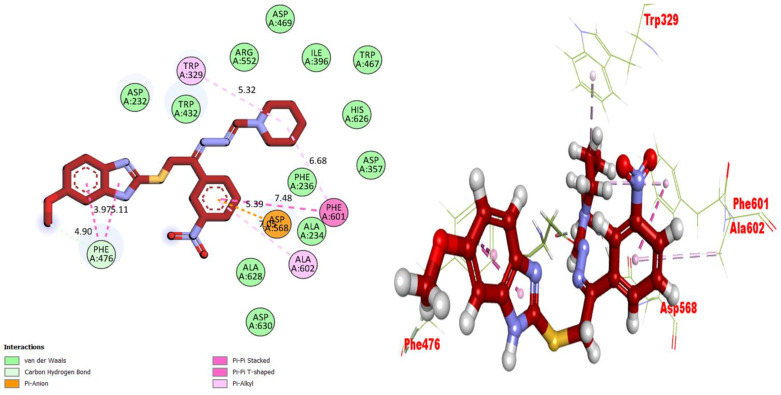
Representations of the protein–ligand interaction (PLI) profile of compound **4** in the BuChE complex.

**Figure 9:**
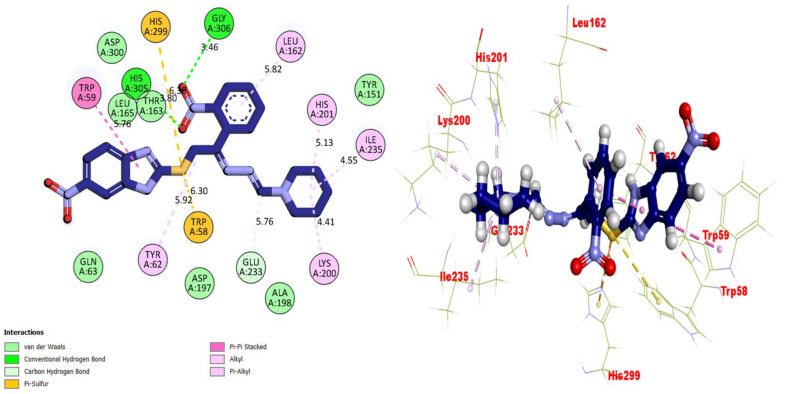
Representations of the protein–ligand interaction (PLI) profile of compound **13** in the AChE complex.

**Figure 10:**
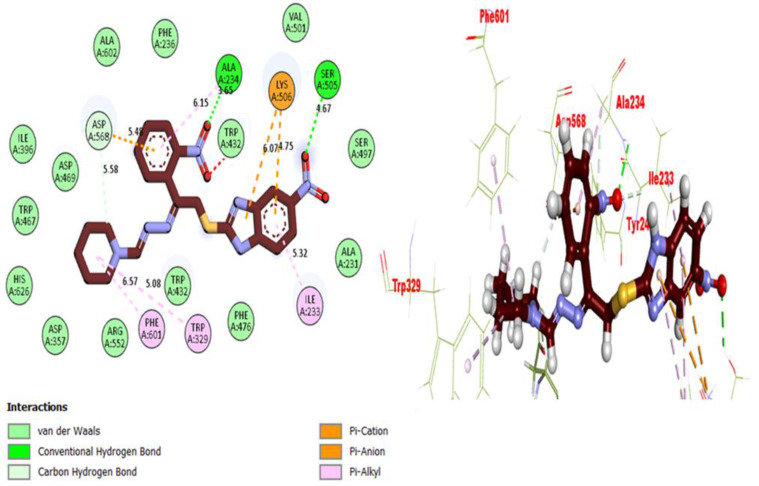
Representations of the protein–ligand interaction (PLI) profile of compound **13** in the BuChE complex.

Molecular docking studies were performed for the compounds (bioactive moieties) in the synthesized series. Different software packages were used in order to sketch out the molecules in the protein complexes, namely, the auto dock tool, Pymol (2.5.2) and the Discovery studio visualizer (DSV). Our research groups have already worked on a similar package while using varied heterocyclic moieties [33,34,35]. Ligands were docked in the same places on the amino acids, where co-crystallized ligands were present in order to check the binding interactions of the synthesized moieties. For the purpose of docking, proteins were downloaded from the RCSB protein data bank (PDB) using the code 1ACL for AChE and 1P0P for BuChE, and both the proteins and ligand molecules were prepared in the DSV and the files were transferred to the auto dock tool, where polar hydrogens and charges were added, and the configuration files were saved in PDBQT format. Upon completion of these steps, the docking procedures were run and all the poses were visualized. After the successful completion of the procedures, nine different poses were obtained for each molecule along with binding affinities. Among these, the poses with very negative values (binding affinity) were selected. All the biologically potent molecules were docked into the binding pocket of the corresponding protein (Figure 3, Figure 4, Figure 5, Figure 6, Figure 7, Figure 8, Figure 9 and Figure 10). Docking scores and binding distances were also calculated (Table 2). Two- and three-dimensional binding visualizations are shown in the figures below. 

#### 2.3.2. Docking Study for Benzimidazole-Based Piperidine Analogs 

The selected compounds (bioactive moieties) in the synthesized series were subjected to molecular docking experiments. To draw out the molecules in the protein complexes, various software packages were employed, including the auto dock tool, PyMol and Discovery studio visualizer (DSV). Our research groups have previously worked on a comparable package with other heterocyclic moieties [33,34,35]. This type of study is used to investigate protein–ligand interactions (PLIs) and is one of the simplest ways to detect the fundamental interaction of molecules with a certain amino acid in a protein complex. Ligands were docked in the same amino acid position as the co-crystallized ligand to test the binding interactions of the synthesized moieties. Proteins were retrieved using the code 1ACL for AChE and 1P0P for BuChE from the RCSB protein data bank (PDB) for docking purposes, and both protein and ligand molecules were created in the DSV before being transferred to the auto dock tool, where polar hydrogen and charges were added and configuration files were produced in PDBQT format. After completing these steps, the docking operations were conducted, and all the positions were visualized. After successfully completing the task, nine different poses for each molecule were obtained along with binding affinities (Table 3). Among them, the pose with the most negative value (binding affinity) was chosen for binding visualization. All the potent molecules were docked into the binding pocket of the corresponding protein. Docking scores and binding distances were also calculated. Two- and three-dimensional representations are shown in the figures below. 

### 2.4. ADME Analysis 

**Figure 11:**
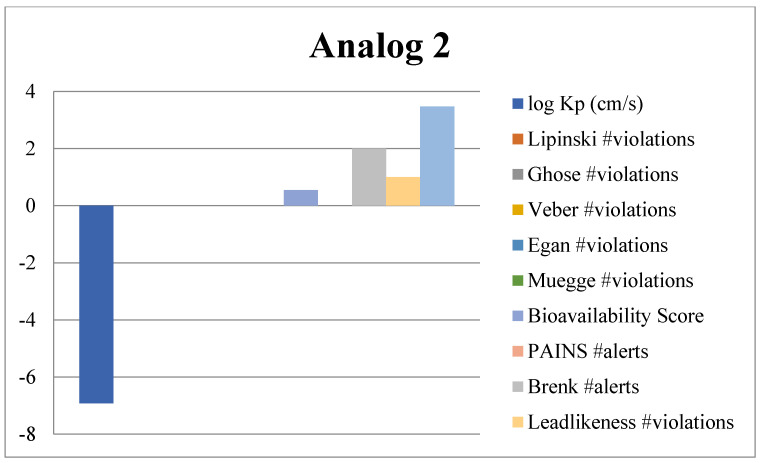
ADME predictions of analog 2.

**Figure 12:**
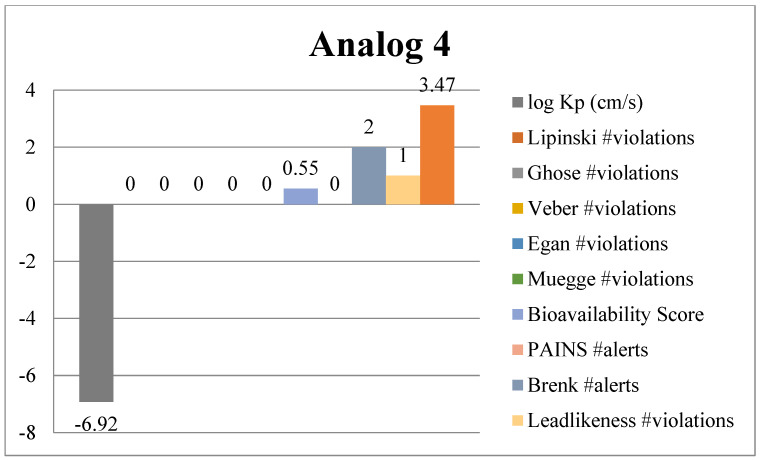
ADME predictions of analog 4.

**Figure 13:**
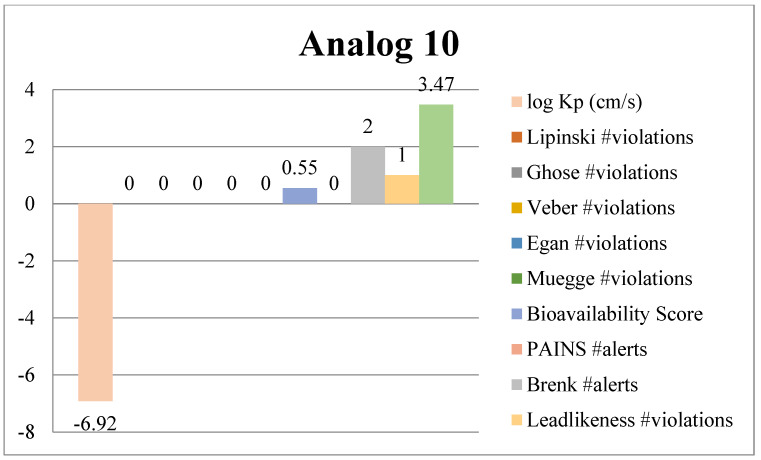
ADME predictions of analog 10.

**Figure 14:**
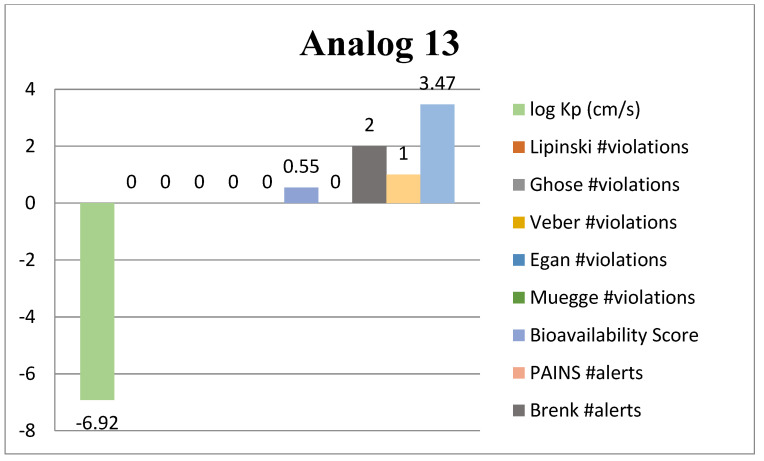
ADME predictions of analog 13.

Absorption, Distribution, Metabolism and Excretion (ADME) analysis was conducted to evaluate the drug likeness properties of the synthesized potent benzimidazole-bearing pyrrole and piperidine hybrid derivatives using an online tool, Swiss ADME (Web online server). Significant outcomes of the ADME analysis further enhanced the molecular docking and biological activity studies. The ADME analysis also showed that marketed drugs violate drug likeness, whereas the potent compounds of the current novel series were reported with violations. These rules include PAINS, log Kp, lead likeness, Brenk alert, Veber, Ghose, Lipinski, Egan, bioavailability score and Muegge violations. The active compounds 2, 4, 10 and 13 of the novel series exhibited excellent inhibitory potency and showed remarkable drug likeness properties, which are illustrated in Figure 11, Figure 12, Figure 13 and Figure 14.

## 3. Material and Methods

### 3.1. General Information 

All the chemicals were purchased from Sigma Aldrich^®^, St. Louis, MO, USA. For NMR experiments, an Advance Bruker AM-500 MHz instrument was used. To obtain high-resolution electron impact mass spectra (HR-EIMS), a Finnigan MAT-311A (Bremen, Germany) mass spectrometer was used. Thin-layer chromatography (TLC) was performed on pre-coated silica gel aluminum plates (Kieselgel 60, 254, E. Merck, Darmstadt, Germany). To visualize the chromatograms, UV at 254 and 365 nm was used. The solvent that was used for the reaction was of analytical grade (Merck, Germany), while commercial solvents (for washing purposes) were purchased from a local supplier and were distilled before use.

### 3.2. General Procedure for the Synthesis of Benzimidazole-Based Pyrrole/Piperidine Derivatives

Targeted pyrrole/piperidine derivatives (**1**–**26**) based on benzimidazole were afforded via several steps. 2-Marcaptobenzimidazole (I, 1 equivalent) was treated with variously substituted phenacyl bromides (1 equivalent) in ethanol (10 mL) and triethylamine (catalyst), and the resulting reaction mixture was then refluxed for 4 h to offer S-substituted benzimidazole (II, 1 equivalent), which was then further redissolved in hydrazine hydrate (5 mL) solution while being agitated in acetic acid (10 mL). The solvent was evaporated after 3 h of refluxing, and the resultant solid residue (III) underwent two distinct reactions. Substrate (III, 1 equivalent) was first placed in ethanol (10 mL) and triethylamine (catalyst), and then 1H-pyrrole-3-carbaldehyde (1 equivalent) was added. For seven hours, the reaction mixture was refluxed and stirred to form the desired pyrrole derivatives (**1**–**13**) based on benzimidazole. Additionally, for the benzimidazole-based piperidine derivatives (**14**–**26**), the intermediate (III, 1 equivalent) was again reacted with piperidine-1-carbaldehyde (1 equivalent) in ethanol (10 mL) along with catalytic amounts of triethylamine.

### 3.3. Spectral Analysis (***1***–***26***)

#### 3.3.1. 2-(((E)-2-(((E)-(1H-pyrrol-3-yl)methylene)hydrazono)-2-(p-tolyl)ethyl)thio)-5-methoxy-1H-benzo[d]imidazole (**1**) 

Yield: 62%, ^1^H-NMR (600 MHz, DMSO-*d*_6_): δ 7.71 (d, *J* = 7.8 Hz, 1H, Ar-H), 7.51 (m, *J* = 7.6, 1.7 Hz 1H, NH), 7.49 (m, *J* = 7.6 Hz, *J* = 1.3 Hz, 1H, Ar-H), 7.30 (d, *J* = 7.9 Hz, 2Hs, Ar-H), 6.99 (d, *J* = 7.4 Hz, 1H, Ar-H), 6.8 (m, *J* = 7.8 Hz, *J* = 3.1, 2Hs, NH), 6.1 (t, *J* = 7.3 Hz, 1H, Ar-H), 5.1 (s, 2H, NH), 3.79 (s, 3H), 3.20 (s, 2H), 2.30 (s, 3H). ^13^C-NMR (150 MHz, DMSO-*d*_6_): 167.9, 155.7, 145.7, 139.2, 138.9, 133.0, 130.4, 129.4, 128.4, 128.4, 127, 127, 122.9, 120.4, 119.8, 116.2, 115, 108.2, 55.8, 55.8, 37.7, 37.7, 21.17, 21.17. HREI-MS: *m*/*z* calcd for C_22_H_21_N_5_OS [M]^+^ 403.1698, found 403.1707.

#### 3.3.2. 2-(((E)-2-(((E)-(1H-pyrrol-3-yl)methylene)hydrazono)-2-(4-methoxyphenyl)ethyl)thio)-5-methoxy-1H-benzo[d]imidazole (**2**)

Yield: 60%, ^1^H-NMR (600 MHz, DMSO-*d*_6_): δ 7.9 (d, *J* = 7.4 Hz, 2Hs, Ar-H), 7.52 (s, 1H, Ar-H), 7.49 (dd, *J* = 7.8, 1.8 Hz, 1H, Ar-H), 7.14 (s, 1H, Ar-H), 7.08 (d, *J* = 7.3 Hz, 1H, Ar-H), 6.91 (d = 7.2 Hz, 1H, Ar-H), 5.9 (d, *J* = 7.3 Hz, 1H, Ar-H), 5.1 (s, 2H, NH), 3.70 (s, 3H), 3.16 (s, 2H). ^13^C-NMR (150 MHz, DMSO-*d*_6_): 164.7, 156.4, 149.3, 147.4, 140.4, 139.5, 131.3, 131.3, 129.2, 129.2, 127.3, 127.3, 122.3, 120.4, 119.4, 116.2, 111.2, 108.1, 100.4, 55.2, 37.3, 21.5. HREI-MS: *m*/*z* calcd for C_22_H_21_N_5_OS [M]^+^ 419.1698, found 419.1707. 

#### 3.3.3. 2-(((E)-2-(((E)-(1H-pyrrol-3-yl)methylene)hydrazono)-2-([1,1′-biphenyl]-4-yl)ethyl)thio)-5-methoxy-1H-benzo[d]imidazole (**3**)

Yield: 64%, ^1^H-NMR (600 MHz, DMSO-*d*_6_): δ 12.11 (s, 1H, NH), 8.78 (d, *J* = 7.4 Hz, 2H, Ar-H), 8.05 (d, *J* = 7.4 Hz, 2H, Ar-H), 7.93 (d, *J* = 7.5 Hz, 2H, Ar-H), 7.87 (d, *J* = 7.3 Hz, 2H, Ar-H), 7.72 (s, 1H), 7.49 (d, *J* = 7.6 Hz, 1H, Ar-H), 7.40 (m, *J* = 7.6, 1.3 Hz, 1H, Ar-H), 7.19 (s, 1H, Ar-H), 7.08 (d, *J* = 7.4 Hz, 1H, Ar-H), 7.03 (m, *J* = 7.4, 1.3 Hz, 2H, Ar-H), 6.92 (d, *J* = 7.4 Hz), 6.84 (s, 2H, NH), 4.80 (s, 3H), 3.78 (s, 2H). ^13^C-NMR (150 MHz, DMSO-*d*_6_): 164.5, 156.5, 149.5, 147.2, 143.2, 140.7, 139.8, 132.7, 131.5, 129.5, 129.4, 129.4, 129.4, 128.4, 128.4, 127.8, 127.8, 127.5, 122.5, 120.6, 119.6, 116.6, 111.6, 108.6, 100.6, 55.5, 37.4; HREI-MS: *m*/*z* calcd for C_27_H_23_N_5_OS [M]^+^ 465.1698, found 467.1707.

#### 3.3.4. 2-(((E)-2-(((E)-(1H-pyrrol-3-yl)methylene)hydrazono)-2-(2-methoxyphenyl)ethyl)thio)-5-methoxy-1H-benzo[d]imidazole (**4**)

Yield: 61%, ^1^H-NMR (600 MHz, DMSO-*d*_6_): δ 7.61 (m, *J* = 7.6, 1.3 Hz, 1H, Ar-H), 7.49 (s, 1H, Ar-H), 7.46 (d, *J* = 7.4, 1H, Ar-H), 7.42 (m, *J* = 7.8, 1.4 Hz, 1H, Ar-H), 7.29 (m, *J* = 7.4, 1.3 Hz, 1H, Ar-H), 7.16 (s, 1H, Ar-H), 7.01 (m, *J* = 7.8, 1.7 Hz, 1H, Ar-H), 6.9 (d, *J* = 7.4, 1.3, 2H, Ar-H), 6.0 (d, *J* = 7.4, 1H, Ar-H), 5.0 (s, 2H, NH), 3.90 (s, 3H), 3.12 (s, 2H). ^13^C-NMR (150 MHz, DMSO-*d*_6_): 147.2, 122.7, 119.3, 139.3, 131.3, 120.5, 108.4, 156.4, 160.4, 100.4, 116.4, 117.3, 111.4, 111.4, 131.6, 132.1, 121.1, 38.1, 55.9, 55.9, 149.3, 164.0 HREI-MS: *m*/*z* calcd for C_22_H_21_N_5_OS [M]^+^ 419.1698, found 419.1707. 

#### 3.3.5. 2-(((E)-2-(((E)-(1H-pyrrol-3-yl)methylene)hydrazono)-2-([1,1′-biphenyl]-4-yl)ethyl)thio)-1H-benzo[d]imidazole (**5**) 

Yield: 65%, ^1^H-NMR (600 MHz, DMSO-*d*_6_): δ 7.90 (d, *J* = 7.4, 2H, Ar-H), 7.88 (d, *J* = 7.6, 2H, Ar-H), 7.61 (m, *J* = (7.7, 1.3 Hz, 2H, Ar-H), 7.5 (m, *J* = 7.6, 1.3 Hz, 2H, Ar-H), 7.49 (m, *J* = 7.5, 1.3, 2H, Ar-H), 7.43 (m, *J* = 7.5, 1.7 Hz, 2H, Ar-H), 7.24 (m, *J* = 7.5, 1.3 Hz, 2H, Ar-H), 6.8 (m, 2H, Ar-H), 6.2 (d, *J* = 7.3 Hz, 1H, ArH), 5.2 (s, 2H, NH), 3.14 (s, 2H). ^13^C-NMR (150 MHz, DMSO-*d*_6_): 147.2, 143.2, 140.7, 138.8, 138.8, 132.8, 129.8, 128.2, 128.2, 127.7, 127.7, 123.8, 123.8, 122.5, 120.5, 119.7, 115.6, 115.6, 108.4. HREI-MS: *m*/*z* calcd for C_26_H_21_N_5_OS [M]^+^ 45,355.198, found 455.5507. 

#### 3.3.6. 2-(((E)-2-(((E)-(1H-pyrrol-3-yl)methylene)hydrazono)-2-(3-nitrophenyl)ethyl)thio)-1H-benzo[d]imidazole (**6**)

Yield: 69%, ^1^H-NMR (600 MHz, DMSO-*d*_6_): δ 8.54 (m, *J* = 7.8, 1.4 Hz, 1H, Ar-H), 8.36, (m, *J* = 7.8, 1.3 Hz, 1H, Ar-H), 8.16 (m, *J* = 7.8, 1.2 Hz, 1H, Ar-H, 7.80 (m, *J* = 7.4, 1.2 Hz, 1H, Ar-H), 7.61 (m, *J* = 7.4, 1.2 Hz, 2H, Ar-H), 7.51 (s, 1H), 7.23 (m, *J* = 7.1, 1.7 Hz, 2H, Ar-H), 6.8 (d, *J* = 7.3 Hz, 1H, Ar-H), 6.7 (t, *J* = 7.3 Hz, 1H, Ar-H), 5.9 (d, *J* = 7.5 Hz, 1H, Ar-H), 3.14 (s, 1H). ^13^C-NMR (150 MHz, DMSO-*d*_6_): 168.0, 156.3, 148.0, 139.0, 138.7, 134.8, 134.6, 132.9, 132.9, 129.6, 124.4, 123.7, 122.2, 120.8, 119.7, 115.6, 115.6, 108.9, 39.1. HREI-MS: *m*/*z* calcd for C_20_H_16_N_6_O_2_S [M]^+^ 404.198, found 405.5507.

#### 3.3.7. 2-(((E)-2-(((E)-(1H-pyrrol-3-yl)methylene)hydrazono)-2-(p-tolyl)ethyl)thio)-1H-benzo[d]imidazole (**7**)

Yield: 72%, ^1^H-NMR (600 MHz, DMSO-*d*_6_): δ 7.89 (d, *J* = 7.8 Hz, 2H, Ar-H), 7.86 (d, *J* = 7.9 Hz, 2H, Ar-H), 7.60 (d, *J* = 7.7 Hz, 2H, Ar-H), 7.56 (m, *J* = 7.4 Hz, 1.9 Hz, 2H, Ar-H), 7.54 (m, *J* = 7.8 Hz, 1.9 Hz, 2H, Ar-H), 7.52 (s, 1H, Ar-H), 7.44 (m, *J* = 7.6 Hz, 1.8 Hz, 1H, Ar-H), 7.24 (d, *J* = 7.9 Hz, 2H, Ar-H). ^13^C-NMR (150 MHz, DMSO-*d*_6_): 164.7, 149.5, 147.8, 143.8, 140.9, 138.7, 138.7, 132.7, 129.8, 129.8, 129.3, 129.3, 128.1, 128.1, 127.8, 127.8, 127.7, 123.1, 123.1, 122.9, 120.7, 119.7, 115.2, 115.2, 108.2 HREI-MS: *m*/*z* calcd for C_21_H_19_N S [M]^+^ 405.198, found 405.5507.

#### 3.3.8. 2-(((E)-2-(((E)-(1H-pyrrol-3-yl)methylene)hydrazono)-2-(2-methoxyphenyl)ethyl)thio)-1H-benzo[d]imidazole (**8**)

Yield: 70%, ^1^H-NMR (600 MHz, DMSO-*d*_6_): δ 7.60 (m, *J* = 7.7, 1.5 Hz, 2H, Ar-H), 7.57 (m, *J* = 7.6, 1.3 Hz, 1H, Ar-H), 7.52 (s, 1H, Ar-H), 7.43 (m, *J* = 7.4, 1.3 Hz, 1H, Ar-H), 7.26 (m, *J* = 7.6, 1.7 Hz, 2H, Ar-H), 6.99 (m, *J* = 7.6, 1.9 Hz, 1H, Ar-H), 6.7 (m, *J* = 7.8, 1.6 Hz, 1H, Ar-H), 6.2 (d, *J* = 7.6 Hz, 1H, Ar-H), 5.0 (s, 2H, NH), 3.86 (s, 2H), 3.20 (s, 3H). ^13^C-NMR (150 MHz, DMSO-*d*_6_): 164.5, 160.8, 149.9, 147.2, 138.7, 138.7, 132.1, 131.8, 123.7, 123.7, 122.7, 121.8, 120.7, 119.6, 117.5, 115.2, 115.6, 111.7, 108.6. HREI-MS: *m*/*z* calcd for C_21_H_19_N S [M]^+^ 389.138, found 389.1507.

#### 3.3.9. 2-(((E)-2-(((E)-(1H-pyrrol-3-yl)methylene)hydrazono)-2-(4-methoxyphenyl)ethyl)thio)-1H-benzo[d]imidazole (**9**)

Yield: 62%, ^1^H-NMR (600 MHz, DMSO-*d*_6_): δ 7.89 (d, *J* = 7.8 Hz, 2H, Ar-H), 7.61 (m, *J* = 7.6, 1.5 Hz, 2H, Ar-H), 7.23 (m, *J* = 7.9, 1.9 Hz, 2H, Ar-H), 7.10 (d, *J* = 7.5 Hz, 1H, Ar-H), 6.8 (m, *J* = 7.6, 2.1 Hz, 2H, Ar-H), 6.0 (d, *J* = 7.6 Hz, 1H, Ar-H), 5.0 (s, 2H, NH, Ar-H), 3.90 (s, 3H), 3.20 (s, 2H). ^13^C-NMR (150 MHz, DMSO-*d*_6_): 164.8, 162.7, 149.5, 147.7, 138.8, 138.8, 128.8, 128.8, 126.2, 123.1, 123.1, 122.6, 120.5, 119.7, 115.4, 115.4, 114.6, 114.6, 108.4, 55.6, 37.8 HREI-MS: *m*/*z* calcd for C_20_H_15_N_7_O_4_ S [M]^+^ 449.038, found 449.94. 

#### 3.3.10. 2-(((E)-2-(((E)-(1H-pyrrol-3-yl)methylene)hydrazono)-2-(3-nitrophenyl)ethyl)thio)-5-nitro-1H-benzo[d]imidazole (**10**)

Yield: 68%, ^1^H-NMR (600 MHz, DMSO-*d*_6_): δ 8.54 (s, 1H, Ar-H) 8.40 (s, 1H, Ar-H), 8.36 (d, *J* = 7.5, 2.8 Hz, 1H, Ar-H), 8.17 (m, *J* = 7.9, 3.1 Hz, NH), 8.12 (m, *J* = 7.3, 1.8 Hz, 1H, Ar-H), 7.80 (t, *J* = 7.5 Hz, 1H, Ar-H), 7.66 (d, *J* = 7.9 Hz, 1H, NH), 7.55 (s, 1H, Ar-H), 6.7 (m, *J* = 7.5, 2.1 Hz, 2H, Ar-H), 6.1 (d, *J* = 7.6, 1H, Ar-H), 5.1 (s, 2H, NH, Ar-H), 3.18 (s, 2H). ^13^C-NMR (150 MHz, DMSO-*d*_6_): 171.7, 164.5, 149.5, 147.4, 145.4, 142.5, 137.3, 132.2, 132.2, 131.2, 131.2, 126.1, 125.3, 122.8, 120.3, 119.7, 118.9, 116.2, 112.8, 109.2, 45.7. HREI-MS: *m*/*z* calcd for C_20_H_15_N_7_O_4_ S [M]^+^ 449.038, found 449.94. 

#### 3.3.11. 2-(((E)-2-(((E)-(1H-pyrrol-3-yl)methylene)hydrazono)-2-([1,1′-biphenyl]-4-yl)ethyl)thio)-5-nitro-1H-benzo[d]imidazole (**11**)

Yield: 67%, ^1^H-NMR (600 MHz, DMSO-*d*_6_): δ 8.40 (s, 1H, Ar-H), 8.1 (d, *J* = 7.6 Hz, 1H, Ar-H), 7.88 (d, *J* = 7.6 Hz, 2H, Ar-H), 7.86 (d, *J* = 7.8 Hz, 2H, Ar-H), 7.70 (d, *J* = 7.6 Hz, 1H, Ar-H), 7.54 (m, *J* = 7.5, 2.8 Hz, 2H, Ar-H), 7.53 (m, *J* = 7.7, 1.8 Hz, 2H, Ar-H), 7.54 (s, 1H, Ar-H), 7.41 (m, 7.8, 1.9 Hz, 2H, Ar-H), 6.5 (m, *J* = 7.4, 1.8 Hz 2H, Ar-H), 6.2 (d, *J* = 7.5 Hz, 1H, Ar-H), 5.1 (s, 2H, NH Ar-H), 3.18 (s, 2H). ^13^C-NMR (150 MHz, DMSO-*d*_6_): 164.5, 149.4, 147.7, 145.6, 144.6, 143.4, 140.5, 139.7, 132.8, 129.8, 129.7, 129.8, 129.7, 128.8, 128.6, 127.7, 127.7, 127.7, 122.9, 120.7, 119.7, 118.7, 116.7, 112.6, 108.7, 37.5. HREI-MS: *m*/*z* calcd for C_26_H_20_N_6_O_2_ S [M]^+^ 480.138, found 480.59. 

#### 3.3.12. 2-(((E)-2-(((E)-(1H-pyrrol-3-yl)methylene)hydrazono)-2-(p-tolyl)ethyl)thio)-5-methoxy-1H-benzo[d]imidazole (**12**)

Yield: 65%, ^1^H-NMR (600 MHz, DMSO-*d*_6_): δ 8.38 (s, 1H, Ar-H), 8.12 (d, *J* = 7.5 Hz, 1H, Ar-H), 7.93 (d, *J* = 7.4 Hz, 2H, Ar-H), 7.68 (d, 7.3 Hz, 1H, Ar-H), 7.53 (s, 1H, Ar-H), 7.11 (d, *J* = 7.5 Hz, 1H, Ar-H), 6.7 (m, *J* = 7.6, 1.8 Hz, 2H, Ar-H), 5.9 (d, *J* = 7.5 Hz, 1H, Ar-H), 5.1 (s, 2H, NH, Ar-H), 3.84 (s, 3H), 3.18 (s, 2H). ^13^C-NMR (150 MHz, DMSO-*d*_6_): 171.7, 162.9, 149.4, 147.1, 145.0, 142.5, 137.3, 135.3, 132.2, 126.3, 122.9, 120.4, 118.9, 118.6, 116.1, 114.4, 114.4, 112.9, 109.2, 55.8, 45.6. HREI-MS: *m*/*z* calcd for C_21_H_18_N_6_O_34_S [M]^+^ 434.1, found 434.4. 

#### 3.3.13. 2-(((E)-2-(((E)-(1H-pyrrol-3-yl)methylene)hydrazono)-2-(p-tolyl)ethyl)thio)-5-nitro-1H-benzo[d]imidazole (**13**)

Yield: 70%*,* ^1^H-NMR (600 MHz, DMSO-*d*_6_): δ 8.40 (s, 1H, Ar-H), 8.13 (d, *J* = 7.4 Hz, 1H, Ar-H), 7.74 (d, *J* = 7.5 Hz, 2H, Ar-H), 7.52 (s, 1H, Ar-H), 7.30 (d, *J* = 7.6 Hz, 1H, Ar-H), 6.5 (m, 2H, Ar-H), 5.9 (d, *J* = 7.5 Hz, 1H, Ar-H), 5.1 (s, 2H, NH, Ar-H), 3.18 (s, 2H), 2.84 (s, 3H). ^13^C-NMR (150 MHz, DMSO-d_6_): 147.8, 122.8, 119.7, 164.8, 149.7, 145.5, 144.8, 140.8, 139.7, 131.7, 129.6, 129.6, 127.1, 127.1, 120.1, 118.7, 116.2, 112.2, 108.2, 37.6, 21.5. HREI-MS: *m*/*z* calcd for C_21_H_18_N_6_O_2_S [M]^+^ 418.1, found 418.4.

#### 3.3.14. 2-(((E)-2-([1,1′-biphenyl]-4-yl)-2-(((E)-piperidin-1-ylmethylene)hydrazono)ethyl)thio)-5-methoxy-1H-benzo[d]imidazole (**14**)

Yield: 63%, ^1^H NMR (600 MHz, DMSO-*d*_6_):δ 7.59 (m, 1H, Ar-H), 7.52 (s, 1H, Ar-H), 7.47 (d, *J* = 7.6 Hz, 1H, Ar-H), 7.42 (m, *J* = 7.7, 1.9 Hz 1H, Ar-H), 7.30 (m, *J* = 7.4, 1.7 Hz, 1H, Ar-H), 7.17 (d, *J* = 7.4 Hz 1H, Ar-H), 7.0 (d, *J* = 7.6 Hz, 1H, Ar-H), 7.4 (m, *J* = 7.6, 1.9 Hz, 1H, Ar-H), 5.1 (s, 1H, NH, Ar-H), 3.86 (s, 1H), 3.52 (dd, *J* = 7.6, 1.9 Hz, 3H), 1.60 (dd, 7.8, 1.7 Hz 1H), 1.56 (dd, 7.8, 1.8 Hz 2H). ^13^C-NMR (150 MHz, DMSO-*d*_6_): 164.5, 156.2, 155.1, 147.4, 143.7, 140.8, 139.8, 132.8, 131.3, 129.7, 129.8, 129.6, 129.7, 128.6, 128.6, 127.6, 127.6, 127.6, 116.3, 111.7, 100.9, 48.8, 48.6, 25.6, 25.6, 24.4, 37.6, 55.7. HREI-MS: *m*/*z* calcd for C_28_H_29_N_5_OS [M]^+^ 483.2, found 483.4. 

#### 3.3.15. 5-methoxy-2-(((E)-2-(2-methoxyphenyl)-2-(((E)-piperidin-1-ylmethylene)hydrazono) ethyl)thio)-1H-benzo[d]imidazole (**15**) 

Yield: 66%, ^1^H-NMR (600 MHz, DMSO-*d*_6_): δ 7.60 (m, *J* = 7.6, 1.9 Hz, 1H, Ar-H), 7.51 (s, 1H, Ar-H), 7.45 (d, *J* = 7.6 Hz, 1H, Ar-H), 7.43 (m, *J* = 7.4, 1.6 Hz, 1H, Ar-H), 7.30 (m, *J* = 7.7, 1.9 Hz, 1H, Ar-H), 7.17 (d, *J* = 7.4 Hz, 1H, Ar-H), 7.0 (d, 7.6 Hz, 1H, Ar-H), 7.4 (m, *J* = 7.8, 1.9 Hz, 1H, Ar-H), 5.1 (s, 1H, NH, Ar-H), 3.86 (s, 1H), 3.51 (m, 3H), 1.60 (m, 1H), 1.56 (m, 2H). ^13^C-NMR (150 MHz, DMSO-*d*_6_): 164.7, 160.8, 156.4, 155.6, 147.5, 139. 1, 132.8, 131.7, 131.8, 121.6, 117.4, 116.7, 111.8, 111.9, 100.7, 55.7, 55.7, 48.8, 48.8, 38.7, 25.7, 24.6, 25.8; HREI-MS: *m*/*z* calcd for C_23_H_27_N_5_OS [M]^+^ 437.2, found 437.4. 

#### 3.3.16. 5-methoxy-2-(((E)-2-(4-methoxyphenyl)-2-(((E)-piperidin-1ylmethylene) hydrazono)ethyl)thio)-1H-benzo[d]imidazole (**16**)

Yield: 68%, ^1^H-NMR (600 MHz, DMSO-*d*_6_): δ 7.60 (m, 1H, Ar-H), 7.5 1 (s, 1H, Ar-H), 7.45 (d, *J* = 7.6 Hz, 1H, Ar-H), 7.43 (m, *J* = 7.8, 1.7 Hz 1H, Ar-H), 7.30 (m *J* = 7.7, 1.8 Hz, 1H, Ar-H), 7.17 (d, *J* = 7.4 Hz 1H, Ar-H), 7.0 (d, *J* = 7.6 Hz, 1.4 Hz, 1H, Ar-H), 7.4 (m, *J* = 7.8, 1.9 Hz, 1H, Ar-H), 5.1 (s, 1H, NH, Ar-H), 3.86 (s, 3H), 3.51 (dd, *J* = 7.8, 2.9 Hz, 1H), 1.60 (dd, *J* = 7.7, 1.9 Hz, 1H), 1.56 (m *J* = 7.6, 1.8 Hz, 2H). ^13^C-NMR (150 MHz, DMSO-*d*_6_): 164.5, 162.8, 156.2, 155.1, 147.2, 139.8, 131.3, 128.8, 128.8, 126.4, 116.5, 114.6, 114.6, 111.7, 100.6, 55.6, 55.6, 48.8, 48.9, 37.7, 25.7, 24.2, 25.7. HREI-MS: *m*/*z* calcd for C_23_H_27_N_5_OS [M]^+^ 437.2, found 437.4. 

#### 3.3.17. 5-methoxy-2-(((E)-2-(3-nitrophenyl)-2-(((E)-piperidin-1-ylmethylene)hydrazono)ethyl) thio)-1H-benzo[d]imidazole (**17**)

Yield: 60%, ^1^H NMR (600 MHz, DMSO-*d*_6_):δ 7.93 (d, *J* = 7.6 Hz Ar-H), 7.51 (s, 1H, Ar-H), 7.46 (d, *J* = 7.6 Hz, 1H, Ar-H), 7.17 (d, *J* = 7.4 Hz 1H, Ar-H), 7.08 (d, *J* = 7.5 Hz, 1H, Ar-H), 7.0 (d, *J* = 7.6 Hz, 1H, Ar-H), 5.1 (s, 1H, NH, Ar-H), 3.86 (s, 3H), 3.51 (m, *J* = 7.8, 1.9 Hz, 1H, Ar-H), 3.16 (s, 2H), 1.61 (m, *J* = 7.9, 1.7 Hz, 1H, Ar-H), 1.56 (m, 2H). ^13^C-NMR (150 MHz, DMSO-*d*_6_): 164.8, 156.5, 155.6, 147.8, 139.7, 134.8, 134.8, 131.6, 131.4, 126.6, 125.7, 116.0, 111.7, 100.6, 55.7, 48.8, 48.8, 37.8, 25.6, 25.6, 24.3. HREI-MS: *m*/*z* calcd for C_22_H_24_N_6_O_3_S [M]^+^ 452.2, found 452.4. 

#### 3.3.18. 5-methoxy-2-(((E)-2-(((E)-piperidin-1-ylmethylene)hydrazono)-2-(p-tolyl)ethyl)thio)-1H-benzo[d]imidazole (**18**) 

Yield: 63%, ^1^H-NMR (600 MHz, DMSO-*d*_6_): δ 8.54 (m, 1H, Ar-H), 8.34 (m, 1H, Ar-H), 7.80 (t, 1H, Ar-H), 7.50 (s, 1H, Ar-H), 7.46 (d, *J* = 7.5 Hz, 1H, Ar-H), 7.0 (d, *J* = 7.5 Hz 1H, Ar-H), 5.2 (s, 1H, NH, Ar-H), 3.53 (m *J* = 7.8, 1.9 Hz, 1H, Ar-H), 3.16 (s, 2Hs), 1.61 (m, *J* = 7.7, 1.6 Hz, 1H 1H), 1.56 (m *J* = 7.7, 1.9 Hz, 1H, Ar-H). ^13^C-NMR (150 MHz, DMSO-*d*_6_): 164.5, 156.3, 155.4, 147.6, 140.6, 139.7, 131.5, 131.8, 129.7, 129.7, 127.8, 127.8, 116.6, 111.7, 100.7, 55.7, 48.8, 48.0, 37.8, 25.9, 25.9, 24.6, 21.4. HREI-MS: *m*/*z* calcd for C_23_H_27_N_5_OS [M]^+^ 421.2, found 421.4

#### 3.3.19. 2-(((E)-2-(((E)-piperidin-1-ylmethylene)hydrazono)-2-(p-tolyl)ethyl)thio)-1H-benzo[d]imidazole (**19**) 

Yield: 66%, ^1^H-NMR (600 MHz, DMSO-*d*_6_): δ 7.74 (d, *J* = 7.5 Hz, Ar-H), 7.51 (d, *J* = 7.5, 1H), 7.36 (d, *J* = 7.7 Hz, 1H, Ar-H), 7.16 (d, *J* = 7.4 Hz, 1H, Ar-H), 7.02 (d, *J* = 7.5 Hz, 1H, Ar-H), 5.1 (s, 1H, NH, Ar-H), 3.86 (s, 3H), 3.51 (m, *J* = 7.6, 1.7 Hz, 1H, 2H), 3.16 (s, 2H), 1.60 (m, *J* = 7.6, 1.3 Hz, 1H), 1.54 (m, *J* = 7.2, 1.6 Hz, 1H, 2H). ^13^C-NMR (150 MHz, DMSO-*d*_6_): 164.0, 155.0, 147.1, 140.7, 139.9, 138.9, 138.9, 131.2, 131.0, 129.1, 129.1, 127.0, 127.0, 123.0, 123.0, 116.2, 115.5, 48.7, 48.7, 37.7, 25.7, 25.7, 24.2, 21.3.HREI-MS: *m*/*z* calcd for C_22_H_25_N_5_S [M]^+^ 391.2, found 391.4 

#### 3.3.20. 2-(((E)-2-(3-nitrophenyl)-2-(((E)-piperidin-1-ylmethylene)hydrazono)ethyl)thio)-1H-benzo[d]imidazole (**20**) 

Yield: 65%, ^1^H-NMR (600 MHz, DMSO-*d*_6_): δ 8.54 (d, *J* = 7.5 Hz, 1H, Ar-H), 8.34 (m, 1H, Ar-H), 8.17 (m, 1H, Ar-H), 7.80 (m, 2H, Ar-H), 7.61 (d, *J* = 7.5 Hz, 1H, Ar-H), 7.28 (m, *J* = 7.2, 1.6 Hz 1H, Ar-H), 5.1 (s, 1H, NH Ar-H), 3.51 (m, *J* = 7.2, 1.9 Hz, 2H), 3.15 (s, 2H), 1.60 (m, *J* = 7.2, 1.3 Hz, 1H), 1.56 (m, *J* = 7.3, 1.3 Hz, 2H). ^13^C-NMR (150 MHz, DMSO-*d*_6_): 164.8, 155.0, 147.1, 140.7, 138.9, 138.9, 138.9, 134.3, 134.9, 131.2, 131.0, 126.1, 125.1, 123.0, 123.0, 116.2, 115.5, 48.7, 48.7, 37.7, 25.7, 25.7, 24.2, 21.3. HREI-MS: *m*/*z* calcd for C_21_H_22_N_6_O_2_S [M]^+^ 422.2, found 422.4.

#### 3.3.21. 2-(((E)-2-(2-methoxyphenyl)-2-(((E)-piperidin-1-ylmethylene)hydrazono)ethyl)thio)-1H-benzo[d]imidazole (**21**) 

Yield: 62%, ^1^H-NMR (600 MHz, DMSO-*d*_6_): δ 7.60 (m, 2H, Ar-H), 7.57 (m, *J* = 7.5, 1.6 Hz, 1H), 7.46 (m, *J* = 7.5, 1.3 Hz, 1H, Ar-H), 7.29 (m, *J* = 7.5 Hz, 1.7 Hz, 1H, Ar-H), 7.24 (m, *J* = 7.2 Hz, 1.7 Hz, 2H, Ar-H), 7.0 (d, *J* = 7.5 Hz, 1H, Ar-H), 5.1 (s, 1H, NH), 3.86 (s, 3H), 3.51 (m *J* = 7.2, 1.6 Hz, 2H), 3.16 (s, 2H), 1.60 (m, *J* = 7.2, 1.6 Hz, 1H), 1.54 (m, *J* = 7.2, 1.6 Hz, 2H). ^13^C-NMR (150 MHz, DMSO-*d*_6_): 164.3, 160.3, 155.3, 147.3, 140.7, 138.8, 138.7, 138.7, 132.1, 131.1, 131.1, 123.2, 123.2, 123.2, 121.2, 117.3, 116.3, 115.3, 111.4, 55.4, 48.4, 48.4, 37.4, 25.4, 25.6, 24.1, HREI-MS: *m*/*z* calcd for C_22_H_25_N_5_OS [M]^+^ 407.2, found 407.18. 

#### 3.3.22. 2-(((E)-2-(4-methoxyphenyl)-2-(((E)-piperidin-1-ylmethylene)hydrazono)ethyl)thio)-1H-benzo[d]imidazole (**22**)

Yield: 61%, ^1^H-NMR (600 MHz, DMSO-*d*_6_): δ 7.94 (d, *J* = 7.5 Hz, 1H, Ar-H), 7.60 (m, 2H, Ar-H), 7.7 (m, *J* = 7.3 Hz, 1.2 Hz, 1H, Ar-H), 7.10 (d, *J* = 7.5 Hz, 2H, Ar-H), 5.1 (s, 1H, NH), 3.85 (s, 3Hs), 3.51 (m, *J* = 7.3 Hz, 1.2 Hz, 2H), 3.15 (s, 2H), 1.60 (m, *J* = 7.3 Hz, 1.4 Hz, 1H), 1.56 (m, *J* = 7.9 Hz, 1.2 Hz, 2H). ^13^C-NMR (150 MHz, DMSO-*d*_6_): 164.5, 162.8, 155.1, 147.2, 138.4, 128.5, 128.5, 126.4, 123.4, 123.1, 115.3, 115.3, 114.3, 114.3, 55.8, 48.7, 48.7, 37.7, 25.6, 25.6, 24.1. HREI-MS: *m*/*z* calcd for C_22_H_25_N_5_OS [M]^+^ 407.2, found 407.18 

#### 3.3.23. 2-(((E)-2-([1,1′-biphenyl]-3-yl)-2-(((E)-piperidin-1-ylmethylene)hydrazono)ethyl)thio)-5-nitro-1H-benzo[d]imidazole (**23**) 

Yield: 63%, ^1^H-NMR (600 MHz, DMSO-*d*_6_): δ 8.44 (d, *J* = 7.5 Hz, 1H, Ar-H), 8.14 (d, *J* = 7.5, 1H, Ar-H), 8.09 (s, 1H, Ar-H), 7.96 (m, *J* = 7.4 Hz, 1.8 Hz, 2H, Ar-H), 7.86 (m, *J* = 7.9 Hz, 1.3 Hz 2H, Ar-H), 7.61 (d, *J* = 7.5 Hz, 1H, Ar-H), 7.53 (m, *J* = 7.4 Hz, 1.5 Hz, 2H, Ar-H), 7.5 (m, *J* = 7.4 Hz, 1.3 Hz, 1H, Ar-H), 7.51 (d, *J* = 7.5 Hz, 1H), 7.42 (m, *J* = 7.3 Hz, 1.6 Hz, 1H, Ar-H), 5.1 (s, 1H, NH, Ar-H), 3.51 (m, *J* = 7.1 Hz, 1.2 Hz, 2H), 3.15 (s, 2H), 1.60 (m, *J* = 7.4 Hz, 1.3 Hz 1H), 1.56 (m, *J* = 7.6 Hz, 1.9 Hz, 2H). ^13^C-NMR (150 MHz, DMSO-*d*_6_): 164.4, 155.1, 147.4, 145.3, 144.4, 141.8, 140.9, 139.8, 134.8, 130.1, 127.8, 127.8, 127.8, 118.7, 37.8, 25.8, 25.9, 24.8. HREI-MS: *m*/*z* calcd for C_27_H_26_N_6_O_2_S [M]^+^ 498.2, found 489.68 

#### 3.3.24. 2-(((E)-2-(4-methoxyphenyl)-2-(((E)-piperidin-1-ylmethylene)hydrazono)ethyl)thio)-5-nitro-1H-benzo[d]imidazole (**24**)

Yield: 58%, ^1^H-NMR (600 MHz, DMSO-*d*_6_): δ 8.44 (d, *J* = 7.5 Hz, 1H, Ar-H), 8.10 (d, *J* = 7.5 Hz, 1H, Ar-H), 7.93 (d, *J* = 7.5, 1H), 7.67 (d, *J* = 7.5 Hz, 1H, Ar-H), 7.10 (d, *J* = 7.5 Hz, 2H, Ar-H), 7.51 (s, 2H, Ar-H), 5.1 (s, 1H, NH, Ar-H), 3.85 (s, 3Hs), 3.51 (m, *J* = 7.2 Hz, 1.9 Hz, 2H), 3.15 (s, 2Hs), 1.60 (m, *J* = 7.4 Hz, 1.3 Hz, 1H), 1.56 (m, *J* = 7.2 Hz, 1.6 Hz, 2H). ^13^C-NMR (150 MHz, DMSO-*d*_6_): 164.3, 162.3, 155.1, 147.2, 138.2, 128.7, 128.7, 126.8, 123.8, 123.8, 115.5, 115.5, 114.3, 114.3, 55.9, 48.8, 48.8, 37.6, 25.5, 25.5, 24.6. HREI-MS: *m*/*z* calcd for C_22_H_24_N_6_O_3_S [M]^+^ 452.2, found 452.68 

#### 3.3.25. 5-nitro-2-(((E)-2-(((E)-piperidin-1-ylmethylene)hydrazono)-2-(p-tolyl)ethyl)thio)-1H-benzo[d]imidazole (**25**)

Yield: 71%, ^1^H-NMR (600 MHz, DMSO-*d*_6_): δ 8.40 (s, 1H, Ar-H), 8.12 (d, *J* = 7.5, 1H, Ar-H), 7.73 (d, *J* = 7.5, 2H, Ar-H), 7.51 (s, 2H, Ar-H), 7.69 (d, *J* = 7.5, 1H, Ar-H), 7.27 (d, *J* = 7.5 Hz, 2H, Ar-H), 7.10 (d, *J* = 7.5 Hz, 2H, Ar-H), 5.1 (s, 1H, NH), 3.85 (s, 3H), 3.51 (m, *J* = 7.4 Hz, 1.3 Hz, 2H), 3.15 (s, 2Hs), 2.38 (s, 3H), 1.60 (m, *J* = 7.5 Hz, 1.4 Hz, 1H), 1.56 (m, *J* = 7.3 Hz, 1.4 Hz, 2H). ^13^C-NMR (150 MHz, DMSO-*d*_6_):164.3, 155.3, 147.3, 145.1, 144.1, 140.1, 139.1, 131.1, 129.4, 129.4, 127.4, 127.4, 118.7, 116.2, 112.2, 48.2, 48.2, 37.2, 25.6, 25.6, 21.5, 24.3 HREI-MS: *m*/*z* calcd for C_22_H_24_N_6_O_2_S [M]^+^ 436.2, found 436.68.

#### 3.3.26. 5-nitro-2-(((E)-2-(2-nitrophenyl)-2-(((E)-piperidin-1-ylmethylene)hydrazono)ethyl)thio)-1H-benzo[d]imidazole (**26**) 

Yield: 69%, ^1^H-NMR (600 MHz, DMSO-*d*_6_): δ 8.41 (s, 1H, Ar-H), 8.11 (d, *J* = 7.5, 1H, Ar-H), 8.09 (m, 1H, Ar-H), 7.99 (m, 1H, Ar-H), 7.93 (m, 1H, Ar-H), 7.69 (d, *J* = 7.5, 1H), 7.57 (m, *J* = 7.5 Hz, 1.6 Hz 1H, Ar-H), 7.51 (s, 2H, Ar-H), 5.1 (s, 1H, NH), 3.85 (s, 3H), 3.51 (m, *J* = 7.6 Hz, 1.2 Hz, 2H), 3.15 (s, 2H), 2.38 (s, 3H), 1.60 (m, *J* = 7.5 Hz, 1.4 Hz, 1H), 1.56 (m, *J* = 7.4 Hz, 1.3 Hz, 2H). ^13^C-NMR (150 MHz, DMSO-*d*_6_): 164.6, 155.0, 147.1, 145.0, 144.3, 141.7, 140.8, 139.8, 134.5, 130.2, 130.0, 129.2, 129.2, 127.9, 127.9, 127.6, 127.1, 126.3, 118.6, 116.0, 112.0, 48.7, 48.7, 37.7, 25.7, 24.2, 25.7. HREI-MS: *m*/*z* calcd for C_21_H_21_N_7_O_4_S [M]^+^ 436.2, found 467.68. 

### 3.4. Acetylcholinesterase and Butyrylcholinesterase Inhibition Assays

The enzyme inhibition activities for acetylcholinesterase and butyrylcholinesterase were determined using the method of Ellman et al. [36] with slight modifications. The 62.0 mM sodium phosphate buffer (pH 8.0, 880 μL) used contained 0.002 M of DTNB (5,5′-dithiobis-2-nitrobenzoic acid). AChE and BuChE solutions (40 μL) were added with the test compounds (40 μL) and kept at 25 °C for 10–15 min. The acetylthiocholine or butyrylthiocholine (40 μL) were added just before the initiation of the catalytic reaction. The enzymatic hydrolysis of the substrates encompassing the formation of yellow 5-thio-2-nitrobenzoate anion products was monitored at 412 nm (15 min). The inhibition reactions were repeated in triplicate with a Shimadzu spectrophotometer (Kyoto, Japan).

### 3.5. Assay Protocol for Molecular Docking Study 

This assay was carried out according to our previously reported work [37,38,39]. 

## 4. Conclusions

In the present study, a series of benzimidazole-based pyrrole and piperidine derivatives (**1**–**26**) were synthesized and characterized through ^1^H NMR, ^13^C NMR and HRMS (EI) analysis and evaluated for in vitro AChE and BuChE inhibitory potential. The evaluation results for these synthesized derivatives showed that compound **10** revealed good inhibitory potential against AChE and BuChE, with an IC_50_ even lower than the standard. Moreover, it binds with the active regions of target proteins with good binding affinity. So, based on the in vitro inhibitory and molecular docking results, it can be concluded that compound **10** might be a potential lead candidate for further studies in the development of AChE and BuChE inhibitors.

## Figures and Tables

**Table 1:** Different substituents (R_1_ and R_2_) and in vitro acetylcholinesterase and butyrylcholinesterase inhibitory activities of synthesized benzimidazole-based pyrrole/piperidine derivatives (**1**–**26**).

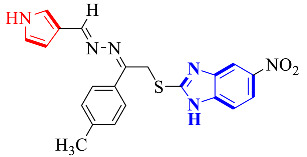				
**S. No.**	**R_1_**	**R_2_**	**AChE Inhibition IC_50_ ± SEM (µM)**	**BuChE Inhibition IC_50_ ± SEM (µM)**
**1**	5-OCH_3_	4-CH_3_	27.11 ± 0.02	31.03 ± 0.11
**2**	5-OCH_3_	4-OCH_3_	20.04 ± 0.15	23.22 ± 0.15
**3**	5-OCH_3_	4-Ph	30.11 ± 0.23	34.06 ± 0.14
**4**	5-OCH_3_	2-OCH_3_	26.22 ± 0.41	29.23 ± 0.80
**5**	H	4-Ph	34.16 ± 0.03	37.21 ± 0.23
**6**	H	3-NO_2_	28.12 ± 0.09	32.14 ± 0.21
**7**	H	4-CH_3_	36.05 ± 0.41	39.55 ± 0.03
**8**	H	2-OCH_3_	21.03 ± 0.11	25.12 ± 0.44
**9**	H	4-OCH_3_	24.16 ± 0.21	28.01 ± 0.13
**10**	5-NO_2_	3-NO_2_	19.44 ± 0.60	21.57 ± 0.61
**11**	5-NO_2_	4-Ph	32.13 ± 0.18	37.76 ± 0.08
**12**	5-NO_2_	4-OCH_3_	23.11 ± 0.35	27.35 ± 0.06
**13**	5-NO_2_	4-CH_3_	27.08 ± 0.25	33.27 ± 0.42
Standard allanzanthane ^a^ drug			16.11 ± 0.33	18.14 ± 0.05 µM
Standard galanthamine ^b^ drug			19.34 ± 0.62	21.45 ± 0.21 µM
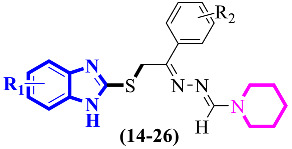				
**S. No.**	**R_1_**	**R_2_**	**AChE Inhibition IC_50_ ± SEM (µM)**	**BuChE Inhibition IC_50_ ± SEM (µM)**
**14**	5-OCH_3_	4-Ph	42.01 ± 0.02	47.03 ± 0.15
**15**	5-OCH_3_	2-OCH_3_	26.03 ± 0.15	29.11 ± 0.14
**16**	5-OCH_3_	4-OCH_3_	28.13 ± 0.23	30.01 ± 0.23
**17**	5-OCH_3_	3-NO_2_	23.09 ± 0.41	27.30 ± 0.50
**18**	5-OCH_3_	4-CH_3_	36.15 ± 0.03	39.11 ± 0.13
**19**	H	4-CH_3_	33.19 ± 0.09	36.22 ± 0.11
**20**	H	3-NO_2_	29.04 ± 0.22	31.25 ± 0.03
**21**	H	2-OCH_3_	30.07 ± 0.41	38.02 ± 0.04
**22**	H	4-OCH_3_	34.02 ± 0.11	34.31 ± 0.12
**23**	5-NO_2_	4-Ph	38.24 ± 0.40	42.27 ± 0.41
**24**	5-NO_2_	4-OCH_3_	32.05 ± 0.14	39.66 ± 0.24
**25**	5-NO_2_	4-CH_3_	35.01 ± 0.25	32.25 ± 0.12
**26**	5-NO_2_	3-NO_2_	22.07 ± 0.13	26.32 ± 0.13
Standard allanzanthane ^a^ drug			20.01 ± 0.12 µM	22.12 ± 0.04 µM
Standard galanthamine ^b^ drug			18.05 ± 0.31 µM	24.25 ± 0.11 µM

Note: ^a^ expressed the standard drug Allanzanthane where ^b^ expressed the standard drug Galanthamine.

**Table 2:** The interaction profiles of the subjected analogs (**2** and **10**) in AChE and BuChE complexes along with docking scores.

Active Analogs	Receptor	Types of Interactions	Distance (A°)	Docking Score
**Analog (2)** in **AChE** complex	GLN-A-63 TRP-A-59 TRP-A-59 TRP-A-62 HIS-A-201 ILE-A-235 LYS-A-200 LEU-A-162 HIS-A-299	H–B Pi–pi stacked Pi–pi stacked Pi–R Pi–S Pi–sigma Pi–R Pi–R Pi–S	5.88 4.31 5.51 4.37 5.58 5.31 5.65 5.81 6.81	−10.50
**Analog (2)** in **BChE** complex	SER-A-505 LYS-A-506 LYS-A-506 TYR-A-243 ALA-A-243 ALA-A-243 ASP-A-568 ASP-A-568 PHE-A-601 TRP-A-329 ILE-A-233	HB Pi–cation Pi–cation Unfavorable A–A HB HB HB C–H Pi–alkyl Pi–alkyl Pi–alkyl	4.67 6.07 4.75 6.09 3.65 6.15 5.48 5.58 6.57 5.08 5.32	−8.97
**Analog (10)** in **AChE** complex	PHE-A-476 PHE-A-476 ALA-A-602 ALA-A-628 ASP-A-630 PHE-A-601 PHE-A-601 ASP-A-568 ASP-A-469 TRP-A-329	Pi–pi T-shaped Pi–pi T-shaped Pi–alkyl Pi–alkyl C–H Pi–pi T-shaped Pi–pi T-shaped C–H C–H Pi–pi T-shaped	5.10 4.27 6.96 7.61 5.35 7.15 7.00 5.55 5.91 6.17	−7.73
**Analog (10)** in **BChE** complex	LEU-A-162 HIS-A-299 TRP-A-58 TRP-A-59 GLY-A-306 HIS-A-305	Pi–alkyl Pi–sulfur Pi–sulfur Pi–pi stacked HB HB	5.80 6.68 6.33 5.56 3.87 3.75	−8.20

**Table 3:** The interaction profiles of the subjected analogs (**4** and **13**) in AChE and BuChE complexes along with docking scores.

Active Analogs	Receptor	Types of Interactions	Distance (A°)	Docking Score
**Analog (4)** in **AchE** complex	ILE-A-235 LYS-A-200 HIS-A-201 LEU-A-162 HIS-A-305 GLY-A-306 HIS-A-299 GLN-A-63 TRP-A-59 TRP-A-59	Pi–R Pi–R Pi–R Pi–R H–B H–B Pi–S H–B Pi–pi stacked Pi–pi stacked	5.59 6.01 5.78 5.83 3.68 4.05 6.87 5.89 4.34 5.36	−9.3
**Analog (4)** in **BuChE** complex	PHE-A-476 PHE-A-476 PHE-A-476 TRP-A-329 PHE-A-601 PHE-A-601 ASP-A-568 ALA-A-602	Pi–pi stacked Pi–pi stacked C–H Pi–R Pi–R Pi–pi stacked Pi–anion Pi–R	5.11 3.97 4.90 5.32 6.68 7.48 5.39 7.05	−8.2
**Analog (13)** in **AchE** complex	TRP-A-59 HIS-A-305 HIS-A-299 GLY-A-306 LEU-A-162 HIS-A-201 ILE-A-235 LYS-A-200 GLU-A-233 TRP-A-58 TYR-A-62	Pi–R Pi–pi stacked H–B Pi–anion H–B Pi–R Pi–R Pi–R C–H Pi–anion Pi–R	5.76 3.80 6.38 3.46 5.82 5.13 4.55 4.41 5.76 6.30 5.92	−7.8
**Analog (13)** in **BuChE** complex	TRP-A-329 PHE-A-601 ASP-A-568 ASP-A-568 ALA-A-234 ALA-A-234 TYR-A-243 LYS-A-506 LYS-A-506 SER-A-505 ILE-A-233	Pi–R Pi–R C–H Pi–anion H–B Unacceptable D–D Pi–anion Pi–anion H–B Pi–R	5.08 6.57 5.58 5.48 6.15 3.65 6.02 6.07 4.75 4.67 5.32	−7.6

## Data Availability

Data is contained within the article.
